# Utility of RECELL for Traumatic Skin Defects: Protocol for a Prospective, Single-Arm Pilot Study

**DOI:** 10.2196/74159

**Published:** 2026-01-09

**Authors:** Naoki Maegawa, Hironobu Konishi, Keisuke Masuda, Akinori Okuda, Hideki Asai, Keita Miyazaki, Yasuyuki Kawai, Hidetada Fukushima

**Affiliations:** 1 Department of Emergency and Critical Care Medicine, Nara Medical University, Shijyou-cho 840, Kashihara, 6348522, Japan, 81 744-22-3051 ext 3443, 81 744-22-5992

**Keywords:** skin grafting, flap surgery, autograft, wound healing, extremity trauma, noncultured skin cell suspension technique, split-thickness skin graft, open fracture

## Abstract

**Background:**

Skin grafting is currently the gold standard for managing traumatic skin defects. However, split-thickness skin grafting, the most common technique, is challenging because it yields a limited amount of skin and creates a new wound at the healthy donor site. RECELL (Avita Medical), a noncultured skin cell suspension technique, yields healing outcomes with comparable duration and quality to skin grafting for burn trauma. Although RECELL is widely used for burn trauma, its use for severe traumatic skin defects is not yet prevalent in Japan.

**Objective:**

This study aims to evaluate the utility of the RECELL skin reconstruction technique in trauma cases involving significant skin defects. Specifically, the primary aim is to validate the efficacy of the RECELL skin reconstruction technique when combined with split-thickness mesh grafting in patients with significant posttraumatic skin defects, with a particular focus on wound closure and scar quality.

**Methods:**

This prospective, single-center, open-label pilot study will enroll patients aged 16 years and older with traumatic limb skin defects ≥160 cm² and sufficient dermal-like tissue. Participants will undergo RECELL-assisted autologous skin grafting with mesh grafts under general anesthesia. Wound healing, scarring (Vancouver Scar Scale), and pain (visual analog scale) will be assessed up to 24 weeks postoperatively. The primary end point is ≥95% reepithelialization at postoperative day 14. Descriptive statistical analyses will be performed to evaluate outcomes and inform future randomized trials.

**Results:**

Patient recruitment began in April 2024 and is currently ongoing in our hospital. As of April 10, 2025, 10 patients had been screened, of whom 4 were enrolled. No adverse events or protocol deviations have been noted.

**Conclusions:**

This study aims to validate the utility of RECELL for skin defects resulting from trauma by assessing wound closure and scar formation, including adverse events, delayed healing, infection, and durability.

## Introduction

Skin grafting is currently the gold standard for managing skin defects [1]. Split-thickness skin grafting, the most common technique, is challenging because it yields a limited amount of skin and creates a new wound at a healthy donor site [2]. For extensive skin defects caused by severe trauma, such as open fractures or degloving injuries, the limited availability of harvested skin often necessitates extended coverage using mesh grafting. However, epithelialization of the meshed area requires a considerable amount of time, and the cosmetic outcome may be less than ideal [3-6]. RECELL (Avita Medical) is a regenerative technique that uses the skin cells of a patient to create a noncultured skin cell suspension for wound healing [7-9]. This suspension is applied to the wound, enabling closure without extensive skin grafting. Although RECELL is widely used for burn trauma, its use for severe traumatic skin defects is not yet prevalent in Japan. To our knowledge, only a few studies have investigated the use of RECELL in patients with extremity trauma [10]. Therefore, this study aimed to evaluate the efficacy of the RECELL skin reconstruction technique in patients with significant posttraumatic skin defects.

While numerous preceding studies have demonstrated the utility of RECELL in the treatment of burn wounds, there are critical differences between burn and traumatic wounds. Burn wounds are typically clean and are primarily characterized by thermally induced vascular damage. In contrast, traumatic wounds, particularly those resulting from crush or degloving injuries, carry a higher risk of contamination and often involve deeper and more complex tissue damage, including damage to blood vessels and subcutaneous structures. However, in large traumatic defects, if a clean and well-vascularized wound bed can be successfully prepared, there is a potential to achieve a higher graft take rate compared to burns, as the peripheral microcirculation may be relatively preserved. Currently, robust data demonstrating the efficacy of RECELL in combination therapy for large traumatic skin defects are lacking. To address this knowledge gap, we sought to clarify the treatment outcomes of layered skin grafting incorporating the RECELL system for traumatic soft tissue defects. Currently, RECELL is not covered by Japanese insurance for the treatment of traumatic skin defects. The findings of this study will aid in the expansion of RECELL technology application in trauma cases. Therefore, the primary aim of this study is to assess the utility and safety of RECELL combined with split-thickness meshed skin grafting in the reconstruction of large posttraumatic skin defects.

## Methods

### Study Design, Participants, and Setting

This is a single-center, open-label, uncontrolled, single-arm, comparative prospective study.

The participants will be patients with open limb wounds transported to our center by ambulance for treatment, with good subcutaneous granulation tissue formation. Furthermore, considering that the area of the skin defect covered by one RECELL 1 kit is 160 cm^2^, we targeted patients with epidermal defects measuring ≥160 cm^2^. Given that the number of such cases transported to our hospital in the past was approximately 5 per year, we decided to target cases over a period of 2 years. The study period will therefore last from April 1, 2024, to March 31, 2026.

### Ethical Considerations

This study will be conducted in compliance with the Declaration of Helsinki and the Clinical Trial Act of the Ministry of Health, Labour, and Welfare in Japan. The investigators will provide each participant with a sufficient explanation of the study and obtain written informed consent to participate. The study protocol has already been approved by the Nara Medical University Certified Review Board (CRB5200002). The trial has been registered on the Japan Registry of Clinical Trials (jRCTs052230203). This study followed the SPIRIT (Standard Protocol Items: Recommendations for Interventional Trials) guidelines (Checklist 1). All study data will be handled confidentially and stored securely. Participants have the right to withdraw consent at any time without penalty, and their participation data will be handled according to the ethical approval guidelines. All adverse events and serious adverse events will be recorded, investigated, and promptly reported to the Nara Medical University Certified Review Board as required by the protocol. This study will be conducted as part of regular treatment and will not involve any compensation to the participants.

### Inclusion and Exclusion Criteria

This study will include patients with skin defects due to trauma (excluding burns caused by flames, excessive heat, or skin exposure to steam or hot water) or skin defects due to skin flaps. Eligible patients must have a skin defect area of ≥160 cm², excluding the hands and face, and sufficient dermal-like tissue formed at the test site. Participants must be aged 16 years or older at the time of consent and provide written informed consent. For those younger than 18 years, written consent will be obtained from their parents or legal guardians. The area to be grafted will be defined as an area considered difficult to treat with standard skin grafting, based on the range of indications for burns in Japan.

The study will exclude patients younger than 16 years; those with hypersensitivity to sodium lactate (washing solution), anesthetics, adrenaline, povidone-iodine, or chlorhexidine; and those with allergies to ingredients derived from pigs. Furthermore, patients will be excluded if they are judged to be unsuitable for study by the principal investigator (or subinvestigator). The face was defined as an excluded affected region due to cosmetic and anatomical complexity that requires specialized reconstructive techniques beyond the scope of this protocol. The selected size of ≥160 cm^2^ was chosen not only because it is the effective coverage area of one standard RECELL kit but also to address the efficacy gap identified in the literature regarding large posttraumatic defects. The exclusion criteria were chosen because, although this study considered skin loss due to trauma, treating limb injuries with skin grafts on the hands is highly likely to lead to significant functional impairment and is therefore generally not recommended. Furthermore, allergic reactions to the medications used were considered a contraindication, and therefore patients who exhibit such reactions will be excluded.

### Surgical Treatment Protocol

The enrolled patients will undergo surgery according to the study protocol. Under general anesthesia, skin grafts will be harvested from healthy skin donor sites (the groin, thigh, or scalp). In addition to the RECELL skin grafts, the necessary amount of mesh skin grafts will be collected. A mesh graft will be applied to the defect site where it is to be placed, and a noncultured cell suspension, previously prepared using RECELL, will be sprayed onto the mesh-grafted area. A nonadherent gauze will be applied to the skin graft, and the gauze will be placed over it for bandage fixation.

### Postsurgical Treatment

The bandaged treatment site will be uncovered 6 to 8 days postoperatively, and the wounds will be evaluated at 24 weeks postoperatively. The administration of antibiotics, anticoagulants, steroid ointments, and any type of dressing material will be permitted. However, the following combined treatments will not be allowed: full-layer skin grafts, negative pressure wound closure therapy used solely for skin graft fixation, and cytotoxic drugs (eg, sulfadiazine silver–containing preparations).

Treatment after completing the protocol may include washing, ointment therapy, and negative pressure wound closure therapy. Treatment will be administered at the discretion of the principal investigator and research associate.

### Study Data Collection

This study will involve a comprehensive collection of the participants’ data. This will be initiated by recording the participants’ background information, including age, sex, height, weight, blood pressure, pulse rate, and body temperature, along with a detailed survey of their current and previous medical histories, as well as any underlying medical conditions present at the time of inclusion in the study.

Subsequently, a series of blood tests, including white blood cell, red blood cell, hemoglobin, hematocrit, and platelet counts, along with coagulation indicators, such as the prothrombin time-international normalized ratio and activated partial thromboplastin time, will be performed. We will also measure the levels of fibrinogen, fibrin degradation products, and a range of biochemical markers, including aspartate aminotransferase and alanine aminotransferase, lactate dehydrogenase, creatine phosphokinase, blood urea nitrogen and creatinine, and C-reactive protein.

For the trauma data, the cause and mechanism of injury will be documented in detail. During the protocol surgery, we will evaluate the recipient site by measuring its long and short diameters and thickness and noting any previous treatments and the method of excision. We will record the magnification of the mesh skin graft and the dimensions of the skin donor site. Photographic records of the recipient sites will be obtained immediately before and after surgery.

Posttreatment follow-up will be performed at 1, 2, 4, 6, 8, 12, and 24 weeks. The tolerance levels applied to the follow-up examination dates are defined in Table 1. During the posttreatment follow-up, we will carefully observe the healing of the recipient site; assess scarring using the Vancouver Scar Scale (VSS) [11]; survey patient satisfaction with the treatment; and observe delayed healing, infections, or scarring requiring surgical intervention. For these evaluations, particularly the photographic records and VSS assessments, the same evaluator will assess each patient whenever possible.

**Table 1.** Schedule of follow-up visits and tolerance levels.

Time point		Tolerance level
**Pretreatment**		
−7 days up to treatment		—^a^
**Day 0**		
Treatment day		—
**Posttreatment**		
After 1 week		±1 day
After 2 weeks		±3 days
After 4 weeks		±5 days
After 6 weeks		±6 days
After 8 weeks		±7 days
After 12 weeks		±10 days
After 24 weeks		±14 days

a Not available.

Finally, pain and concomitant treatments will be evaluated. Pain at both the recipient and donor sites will be evaluated using a visual analog scale (VAS). We also plan to maintain a detailed record of any concurrent treatments, including the names, daily doses, and durations of antibiotics and anticoagulants, as well as the types of dressing materials and steroid ointments used. A CONSORT (Consolidated Standards of Reporting Trials)–style timeline of the planned patient enrollment and assessment is shown in Table 2.

**Table 2.** Timeline of the preoperative and postoperative assessment protocol for this study.

	Pretreatment	Treatment day	After 1 week	After 2 weeks	After 4 weeks	After 6 weeks	After 8 weeks	After 12 weeks	After 24 weeks
Obtaining consent	✓								
Photography: test site		✓	✓	✓	✓	✓	✓	✓	✓
Healing of the test site			✓	✓	✓	✓	✓	✓	✓
Scar assessment (VSS^a^)								✓	✓
Subject satisfaction after treatment								✓	✓
Delayed healing of test site					✓	✓	✓	✓	✓
Presence of wound infection		✓	✓	✓	✓	✓	✓	✓	✓
Scars requiring surgical intervention			✓	✓	✓	✓	✓	✓	✓
Assessment of pain at the test site (VAS^b^)			✓	✓	✓	✓	✓		
Diseases failure			✓	✓	✓	✓	✓		
Concomitant treatment survey			✓	✓	✓	✓	✓	✓	✓

a VSS: Vancouver Scar Scale.

b VAS: visual analog scale.

Five years after the end of the study, the information will be processed so that specific individuals cannot be identified unless the information is matched with other information, and the records will be supplemented.

### Data Analysis

This study is designed as a prospective, single-arm pilot study. Given the lack of robust data on RECELL for large traumatic defects, a formal power calculation is not feasible for the primary end point. Therefore, we aim to enroll 10 patients over 2 years, which will provide the descriptive data necessary to inform the design and power calculation for a subsequent randomized controlled trial.

The primary end point is the achievement of ≥95% reepithelialization at the recipient site, assessed at postoperative day 14 (±3 days tolerance), corresponding to the final planned dressing change within the early healing phase (refer to Table 2). We determined that 14 days was appropriate based on our past case experience and several published studies [12-14]. The secondary end points, including the VSS and VAS for scar assessment, will be primarily evaluated at the final observation point of 24 weeks postoperatively (and also at 12 weeks), as detailed in Table 2.

Descriptive analyses for continuous variables will be presented as the mean and SD. Categorical variables will be presented as counts and percentages. Any missing data will be reported, and the analysis will use a complete case analysis approach, given the pilot nature and short follow-up period. Although this is a single-arm pilot study, exploratory comparisons of the primary outcome (reepithelialization rate) and VSS scores will be made against published historical controls to inform the sample size calculation for future randomized controlled trials. Descriptive analyses, including central tendency (mean and median) and variability, will be performed after data collection is completed.

## Results

Patient recruitment began in April 2024 and is currently ongoing. As of April 10, 2025, 10 patients have been screened, of whom 4 were enrolled. No adverse events or protocol deviations were observed. We expect to complete patient enrollment by March 2026, with a follow-up period of 24 weeks postoperatively. The final data analysis is scheduled to commence in mid-2026.

## Discussion

### Anticipated Findings

The hypothesized key finding of this study is that the combination of RECELL and 3:1 meshed skin grafting will demonstrate both favorable wound healing rates and superior long-term scar quality when compared with historical controls of standard skin grafting techniques for similar large traumatic defects. The management of extensive lower extremity soft tissue and skin loss is often difficult; indeed, several comorbidities should be considered and addressed in these patients [11,15]. The approach is multifactorial and requires commitment from both the surgeon and the patient. Several methods have been developed to address soft tissue and skin coverage of the limbs. For recontraction of large skin defects, the existing literature describes the evaluation, harvesting, transplantation, and management of skin grafting techniques for the lower extremities. Thus, soft tissue defects remain a challenge for orthopedic surgery. Such defects are commonly encountered following orthopedic injuries or infections, and the management of these wounds varies significantly [15,16]. In patients with burns, Elkady et al [17] reported that autologous skin cell suspension (ASCS) with or without split-thickness skin grafting reduced the number of surgical procedures, postoperative complications, and potential cost savings compared with split-thickness skin grafting alone. Carter et al [18] noted that these findings underscore the benefits of integrating ASCS with or without split-thickness skin grafting as a practical approach in burn wound management, providing substantial advantages to both patients and health care institutions. We believe that ASCS with or without split-thickness skin grafting is beneficial for extremity trauma because the wound bed is healthier than that of patients with burns [11,19,20]. However, the role of ASCS with or without split-thickness skin grafting in patients with traumatic skin defects remains unclear. There have been some reports on the use of RECELL in trauma cases [21-24]. The safety profiles were comparable, with similar frequencies of treatment-emergent adverse events of interest observed between the treatment areas. In prior case reports and case series of patients presenting with various nonthermal tissue defect types, ASCS has also shown favorable outcomes related to healing, donor site size, and aesthetic appearance [25-27]. A preliminary study of 10 posttraumatic cases treated with RECELL was conducted in 2020 [9]. This report showed that RECELL could be equivalent to a skin graft with complete healing at 30 days for smaller skin defects (<70 cm²). However, the rate of wound healing decreased to below 13% for larger skin defects (>70 cm²). The authors concluded that RECELL could not be the choice for skin reconstruction of larger traumatic defects and that the best reconstructive option remains traditional split-thickness mesh skin grafts. However, among patients with burns, Holmes et al [28] compared the healing outcomes of RECELL alone (mean area 168.2 cm^2^) and 2:1 meshed skin grafting (mean area 165.0 cm^2^) within-patient allocation of treatments and concluded that both treatment modalities were comparable among 87 patients at 4 weeks postoperatively. Although the target patients differ (trauma and burns) between these studies, the results show conflicting efficacy of RECELL; therefore, it seems premature to investigate the efficacy of RECELL alone in patients with trauma. Therefore, we referred to the prospective, multicenter, randomized trial by Gardien et al [29], who compared mesh grafting alone with grafting with cultured autologous proliferating epidermal cells in patients with burns, found that mesh grafting with cultured epidermal cells was more effective than grafting with mesh alone on postoperative days 5 to 7 in terms of wound closure and scar quality [18]. To investigate the impact of RECELL on early wound closure and scar quality, we designed a treatment protocol for 3:1 split-thickness mesh grafting in combination with RECELL [20,30].

Another key feature of this study is that the participants will not be enrolled unless a good wound bed is prepared. Controlling wound infection is a critical factor that must be addressed in patients with trauma. Kahn et al [31] conducted a randomized open-label trial of RECELL for contaminated diabetic foot gangrene, reporting epithelialization in 66.7% of patients. The key factor influencing this relatively high wound closure rate in diabetic foot gangrene was that the study included patients with skin defects with “adequately vascularized” wounds [31]. As the condition of the wound bed plays a major role in the success or failure of skin grafting, we believe that including this condition in patients with trauma will yield good epithelialization and wound closure.

In the proposed study, we will also investigate postoperative scar formation. However, the observation period will be relatively short (24 weeks). Holmes et al [28] recorded VSS scores for burn injuries with RECELL at 16, 24, and 52 weeks. The authors reported no significant difference between the RECELL and mesh skin grafts throughout the study period. However, we believe that a study period of 24 weeks is sufficient to observe scar formation.

We acknowledge several limitations to this study. First, it is a single-center, open-label pilot study with a small target sample size (n=10), limiting the generalizability of the findings. Second, the lack of a contemporaneous control group means all comparisons with standard care must rely on historical or published data, which introduces inherent bias. However, the data gathered will be essential for calculating the required sample size for a future, adequately powered randomized controlled trial.

### Conclusions

We believe that using RECELL for traumatic skin defects in the extremities will yield better clinical results than using it for patients with burns. With this study protocol, we aim to validate the usefulness of RECELL for skin defects owing to trauma by observing wound closure and scar formation, including adverse events, delayed healing, infection, and durability.

## Supplementary material

10.2196/74159 Checklist 1 SPIRIT checklist.

## Abbreviations

ASCS: autologous skin cell suspension
CONSORT: Consolidated Standards of Reporting Trials
VAS: visual analog scale
VSS: Vancouver Scar Scale

## References

[1] Fuller DA (2016). Split thickness skin graft to lower leg. J Orthop Trauma.

[2] Cho EH, Shammas RL, Carney MJ (2018). Muscle versus fasciocutaneous free flaps in lower extremity traumatic reconstruction: a multicenter outcomes analysis. Plast Reconstr Surg.

[3] Bonaparte JP, Corsten MJ, Odell M, Gupta M, Allen M, Tse D (2013). Management of the radial forearm free flap donor site using a topically applied tissue expansion device. Oral Surg Oral Med Oral Pathol Oral Radiol.

[4] Mosquera C, Weyh A, Malik M, Fernandes R, Bunnell A, Nedrud S (2024). Comparison of the outcomes of split thickness skin graft versus thickness skin graft for closure of the radial forearm free flap donor site: a systematic review. Microsurgery.

[5] Blackburn JH, Boemi L, Hall WW (1998). Negative-pressure dressings as a bolster for skin grafts. Ann Plast Surg.

[6] Legemate CM, Ooms PJ, Trommel N (2020). Course of scar quality of donor sites following split skin graft harvesting: comparison between patients and observers. Wound Repair Regen.

[7] (2023). Instructions for use: RECELL® autologous cell harvesting device. AVITA Medical.

[8] Peirce SC, Carolan-Rees G (2019). ReCell® spray-on skin system for treating skin loss, scarring and depigmentation after burn injury: a NICE medical technology guidance. Appl Health Econ Health Policy.

[9] Moris V, Cristofari S, Stivala A (2020). Recell in post-traumatic cases: preliminary results. J Plast Reconstr Aesthet Surg.

[10] Baryza MJ, Baryza GA (1995). The Vancouver Scar Scale: an administration tool and its interrater reliability. J Burn Care Rehabil.

[11] Khan AA, Khan IM, Nguyen PP (2020). Skin graft techniques. Clin Podiatr Med Surg.

[12] Karlsson M, Elmasry M, Steinvall I, Sjöberg F, Olofsson P, Thorfinn J (2018). Scarring at donor sites after split-thickness skin graft: a prospective, longitudinal, randomized trial. Adv Skin Wound Care.

[13] Kaartinen IS, Kuokkanen HO (2011). Suprathel(®) causes less bleeding and scarring than Mepilex(®) Transfer in the treatment of donor sites of split-thickness skin grafts. J Plast Surg Hand Surg.

[14] Poh Yuen Wen A, Halim AS, Mat Saad AZ, Mohd Nor F, Wan Sulaiman WA (2018). A prospective study evaluating wound healing with sea cucumber gel compared with hydrogel in treatment of skin graft donor sites. Complement Ther Med.

[15] Taylor BC, Triplet JJ, Wells M (2021). Split-thickness skin grafting: a primer for orthopaedic surgeons. J Am Acad Orthop Surg.

[16] Kowal S, Kruger E, Bilir P (2019). Cost-effectiveness of the use of autologous cell harvesting device compared to standard of care for treatment of severe burns in the United States. Adv Ther.

[17] Elkady D, Larson BM, Sharma S (2024). Effectiveness of autologous skin cell suspension in large total body surface area burns: analysis of clinical outcomes and patient charges. J Burn Care Res.

[18] Carter JE, Carson JS, Hickerson WL (2022). Length of stay and costs with autologous skin cell suspension versus split-thickness skin grafts: burn care data from US centers. Adv Ther.

[19] Foster K, Amani A, Carter D (2021). Evaluating health economic outcomes of autologous skin cell suspension (ASCS) for definitive closure in US burn care using contemporary real-world burn center data. J Curr Med Res Opin.

[20] Hu ZC, Chen D, Guo D (2015). Randomized clinical trial of autologous skin cell suspension combined with skin grafting for chronic wounds. Br J Surg.

[21] Bashir MA, Fung V, Kernohan MD, Ragbir M, Ahmed OA (2010). “Z-plasty” modification of ulnar-based fasciocutaneous flap for closure of the radial forearm flap donor defect. Ann Plast Surg.

[22] Wester JL, Pittman AL, Lindau RH, Wax MK (2014). AlloDerm with split-thickness skin graft for coverage of the forearm free flap donor site. Otolaryngol Head Neck Surg.

[23] Sinha UK, Shih C, Chang K, Rice DH (2002). Use of AlloDerm for coverage of radial forearm free flap donor site. Laryngoscope.

[24] Wirthmann A, Finke JC, Giovanoli P, Lindenblatt N (2014). Long-term follow-up of donor site morbidity after defect coverage with Integra following radial forearm flap elevation. Eur J Plast Surg.

[25] Byun SH, Ahn KM, Kim SM, Lee JH (2016). Functional and cosmetic outcome after closure of radial forearm free flap donor defect with porcine collagen membrane. J Craniomaxillofac Surg.

[26] Tane N, Aihara M, Inoue H (1996). The use of artificial dermis on the donor defect of the free forearm flap. J Reconstr Microsurg.

[27] Garcia E, Stone E, Chan LS, Van Vliet M, Garner WL (2014). Donor-site preferences in women during autologous skin grafting. Plast Reconstr Surg.

[28] Holmes Iv JH, Molnar JA, Carter JE (2018). A comparative study of the ReCell® device and autologous spit-thickness meshed skin graft in the treatment of acute burn injuries. J Burn Care Res.

[29] Gardien KL, Marck RE, Bloemen MC (2016). Outcome of burns treated with autologous cultured proliferating epidermal cells: a prospective randomized multicenter intrapatient comparative trial. Cell Transplant.

[30] Carson JS, Carter JE, Hickerson WL (2023). Analysis of real-world length of stay data and costs associated with use of autologous skin cell suspension for the treatment of small burns in U.S. centers. Burns.

[31] Kahn SA, Carter JE, Wilde S, Chamberlain A, Walsh TP, Sparks JA (2024). Autologous skin cell suspension for full-thickness skin defect reconstruction: current evidence and health economic expectations. Adv Ther.

