# Supplementary material for: Utility of RECELL for Traumatic Skin Defects: Protocol for a Prospective, Single-Arm Pilot Study
Source: JMIR Res Protoc. 2026 Jan 9;15:e74159. doi: 10.2196/74159 (PMC12788711; doi:10.2196/74159)
Supplement: Checklist 1 [file resprot-v15-e74159-s001.pdf]

STANDARD PROTOCOL ITEMS: RECOMMENDATIONS FOR INTERVENTIONAL TRIALS

SPIRIT 2013 Checklist: Recommended items to address in a clinical trial protocol and related documents*

Section/item	Item No	Description
**Administrative information**		
Title	1	Utility of RECELL® for Traumatic Skin Defects: A Study
Trial registration	2a	RCTs052230203
	2b	All items from the World Health Organization Trial Registration Data Set
Protocol version	3	Ver1.1 Jan,25. 2024.
Funding	4	Financial Nara medical University<br>Material and financial COSMOTEC Co.Ltd.
Roles and responsibilities	5a	Naoki Maegawa, affiliations, and roles of protocol contributors
	5b	None
	5c	Recell Kits will provided by COSMOTECCo.LT d
	5d	HK,KM,AO,HA and NM :Surgical procedure and data correction<br>KM: Manager of data centre<br>YK: Data analysis<br>NM and HF: Coordinating centre
**Introduction**		

Background and rationale	6a	RECELL® (Avita Medical, Cambridge, UK), a non-cultured skin cell suspension technique, yields result comparable to skin grafting in terms of duration and healing quality. In Japan, the clinical use of RECELL® has become possible for burn trauma but not for skin defects associated with trauma (Line 78-88)
	6b	None
Objectives	7	To evaluate the use of the RECELL® skin reconstruction technique in post-traumatic patients with significant skin defects. (Line89-95)
Trial design	8	Single-center, open-label, uncontrolled, single-arm comparative prospective study（line.99-105）

Methods: Participants, interventions, and outcomes

Study setting	9	Department of Emergency and Critical care Medicine, of Nara medical university.
Eligibility criteria	10	This study will include patients with skin defects due to trauma (excluding burns caused by flames, excessive heat, or skin exposure to steam or hot water) or skin defects due to skin flaps. Eligible patients must have a skin defect area of ≥ 160 cm², excluding the hands and face, and sufficient dermal-like tissue formed at the test site. Participants must be aged ≥ 16 years at the time of consent and provide written informed consent. For those aged < 18 years, written consent will be obtained from their parents or legal guardians. The area to be grafted was determined to be an area that would be difficult to treat with standard skin grafting, based on the range of indications for burns in Japan.(Line114-120)
Interventions	11a	The study will exclude patients aged ≥ 16 years; those with hypersensitivity to sodium lactate (washing solution), anesthetics, adrenaline, povidone–iodine, or chlorhexidine; and those with allergies to ingredients derived from pigs. Furthermore, patients will be excluded if they are judged to be unsuitable study participants by the principal investigator (or sub-investigator). (Line 114-120)
	11b	None (this sturdy is only one surgical procedure)

11c Surgical treatment protocol

The enrolled patients will undergo surgery according to the study protocol. Under general anesthesia, skin grafts will be harvested from healthy skin donor sites (the groin, thigh, or scalp). In addition to the RECELL® skin grafts, the necessary amount of mesh skin grafts will be collected. A mesh graft will be applied to the defect where it is to be placed, and a non-cultured cell suspension, previously prepared using RECELL®, will be sprayed onto the mesh-grafted area. A nonadherent gauze is applied to the skin graft, and the gauze is placed over it for bandage fixation. (Line 134-139)

Postsurgical treatment

The bandaged treatment site will be uncovered 6–8 days postoperatively, and the wounds will be evaluated 24 weeks postoperatively. The administration of antibiotics, anticoagulants, steroid ointments, and any type of dressing material will be permitted. However, the following combined treatments will not be allowed: full-layer skin grafts, negative pressure wound closure therapy used solely for skin graft fixation, and cytotoxic drugs (e.g., sulfadiazine silver–containing preparations).

Treatment after completing the protocol may include washing, ointment therapy, and negative-pressure wound closure therapy. Treatment will be administered at the discretion of the principal investigator and research associate.(Line142-149)

11d The face was excluded due to cosmetic and anatomical complexity which requires specialized reconstructive techniques beyond the scope of this protocol. The ≥160 cm2 was selected not only because it is the effective coverage area of one standard RECELL kit but also to address the efficacy gap identified in the literature regarding large post-traumatic defects. The exclusion criteria were that, although this study considered skin loss due to trauma, treating limb injuries with skin grafts on the hands is highly likely to lead to significant functional impairment and is therefore generally not recommended. Furthermore, allergic reactions to the medications used were a contraindication and therefore excluded. (Line124-131)

Outcomes	12	Photographic records of the recipient sites were obtained immediately before and after surgery. Post-treatment follow-up will be performed at 1, 2,4,6,8,12 and 24 weeks. During the post-treatment follow-up, we carefully observed the healing of the recipient site, assessed scars using the Vancouver Scar Scale (VSS), surveyed patient satisfaction with the treatment, and observed delayed healing, infection, or scar, that required surgical intervention. For these evaluations, particularly the photographic records and VSS assessments, the same evaluator was used for each patient whenever possible.<br>Finally, pain and concomitant treatments were evaluated. Pain at both the recipient and donor sites was evaluated using the Visual Analog Scale (VAS). We also maintained a detailed record of any concurrent treatments, including the names, daily doses, and durations of antibiotics and anticoagulants, as well as the types of dressing materials and steroid ointments used. (Line166-174)
Participant timeline	13	Table1.
Sample size	14	10cases
Recruitment	15	All cases will be in our department.

Methods: Data collection, management, and analysis

Data collection methods	18a	The wound will be examined until 24 weeks after the protocol surgery. Create and save a CRF at the time of examination.
	18b	All Patients will be followed at Our Unit.

Data management	19	Five years after the end of the study, the information will be processed so that specific individuals cannot be identified unless the information is matched with other information, and the records will be supplemented.<br>The collected data will be anonymized and recorded. The correspondence table will be stored in a locked warehouse. Photos and scores will be stored in password-protected folders on a PC.
Statistical methods	20a	Descriptive analyses including central tendency (mean and median) and variability will be performed after data collection has been completed. No interim analysis will be performed.
	20b	No further analyses are planned.
	20c	Cases that deviate from the protocol will be considered as missing.

Methods: Monitoring

Data monitoring	21a	Yayoi Nakamura, Clinical Research Centre Nara Medical University.
	21b	NM and HF
Harms	22	If skin necrosis required re-grafting, the trial was considered a failure; this will also be considered as an endpoint. Descriptive analyses, including central tendency (mean and median) and variability, will be performed after data collection is completed. No interim analyses will be performed.Line 191-222
Auditing	23	All cases<br>Monitoring will be performed initial 1 month and Last time.

Ethics and dissemination

Research ethics approval	24	Nara Medical University Certified Review Board 89 (CRB5200002) (Line89-90)
Protocol amendments	25	Follow the Nara Medical University CRB's default policy.
Consent or assent	26a	NM,HK,KM,AO<br><br>Patients who have obtained or provided written consent. If the patient is aged < 18 years, written consent must also be obtained from a guardian.
	26b<br>No	

Confidentiality	27	Create a code that links the patient's code to his/her personal information so that the patient cannot be identified, and fill out and record the CRF based on the code. The table to be matched will be stored in a locked locker until after the study is completed.
Declaration of interests	28	COSMOTEC Co., Ltd. is the Japanese distributor of the RECELL® device. They provided the RECELL® devices used in this study, which constitutes the conflict of interest. The company had no role in study design, data collection, data analysis, or manuscript preparation.
Access to data	29	All authors
Ancillary and post-trial care	30	Obtain and handle insurance for research
Dissemination policy	31a	We will report at Annual Meeting of Orthopaedic Trauma Association, Journal of Orthopaedic Trauma and Injury.
	31b	NM and HF contributed to the conceptualization and design of the study. The study protocol was primarily drafted by NM, HA, and HF. HK, KM, AO, KM, and YK critically reviewed and revised the manuscript for important intellectual content. All authors have read and approved the final version of the manuscript.(line297-300)
	31c	The organization of the study is shown in jRCTs052230203.
**Appendices**		
Informed consent materials	32	Model consent form and other related documentation given to participants and authorised surrogates
Biological specimens	33	Plans for collection, laboratory evaluation, and storage of biological specimens for genetic or molecular analysis in the current trial and for future use in ancillary studies, if applicable

*It is strongly recommended that this checklist be read in conjunction with the SPIRIT 2013 Explanation & Elaboration for important clarification on the items. Amendments to the protocol should be tracked and dated. The SPIRIT checklist is copyrighted by the SPIRIT Group under the Creative Commons "Attribution-NonCommercial-NoDerivs 3.0 Unported" license.
